# Lung transplantation for idiopathic multicentric Castleman disease: potential efficacy and tolerability of a humanized anti-interleukin-6 receptor monoclonal antibody

**DOI:** 10.1186/s40792-021-01297-2

**Published:** 2021-09-17

**Authors:** Yasuaki Tomioka, Shinji Otani, Shin Tanaka, Kazuhiko Shien, Ken Suzawa, Kentaroh Miyoshi, Hiromasa Yamamoto, Mikio Okazaki, Seiichiro Sugimoto, Masaomi Yamane, Shinichi Toyooka

**Affiliations:** 1 Department of Thoracic Surgery, Organ Transplant Center, Okayama University Hospital, Okayama, Japan; 2 Department of Thoracic Surgery, Okayama University Hospital, Okayama, Japan; 3 Department of Cardiovascular and Thoracic Surgery, Ehime University Medical School, 454 Shizugawa, Toon, 791-0295 Japan

**Keywords:** Lung transplantation, Castleman disease, IL-6, Tocilizumab

## Abstract

**Background:**

Idiopathic multicentric Castleman disease (iMCD) is a rare polyclonal lymphoproliferative disease caused by the overrepresentation of interleukin-6 (IL-6). Tocilizumab (TCZ) is a humanized monoclonal antibody that binds to the IL-6 receptor and is approved for the treatment of iMCD. The efficacy and tolerability of TCZ in patients with iMCD undergoing lung transplantation (LTx) remain unknown.

**Case presentation:**

We present the case of a 48-year-old iMCD patient with end-stage lung disease (ESLD) who was successfully treated with cadaveric single-LTx. Intravenous TCZ was used to stabilize the iMCD patient every 2 weeks, except for withdrawal immediately after LTx. At 32 month post-transplant, the patient remained asymptomatic without evidence of rejection, development of de novo donor-specific antibody (DSA), and recurrent iMCD in the native lung.

**Conclusions:**

Single-LTx can be a feasible treatment option for ESLD caused by iMCD. TCZ can be used safely and may be beneficial in recipients with iMCD, and TCZ in combination with usual immunosuppression can be helpful in stabilizing iMCD patients pre- and post-LTx.

## Background

Idiopathic multicentric Castleman disease (iMCD) is an uncommon polyclonal lymphoproliferative disorder characterized by the elevated levels of interleukin-6 (IL-6) [1–3]. Although iMCD is frequently associated with lung parenchymal involvement, there have been only two reports of lung transplantation (LTx) with a short follow-up for the treatment of end-stage lung disease (ESLD) secondary to iMCD [4, 5]. Tocilizumab (TCZ; Actemra®, Roche/Genentech, San Francisco, CA, USA) is a recombinant humanized monoclonal antibody that binds to the IL-6 receptor and is approved for the treatment of iMCD [6, 7]. However, the efficacy and tolerability of TCZ in iMCD patients undergoing LTx remain unclear. Herein, we report the case of an iMCD patient with ESLD who was treated with TCZ and later successfully underwent cadaveric single-LTx with 32 month follow-up.

## Case presentation

**Fig. 1:**
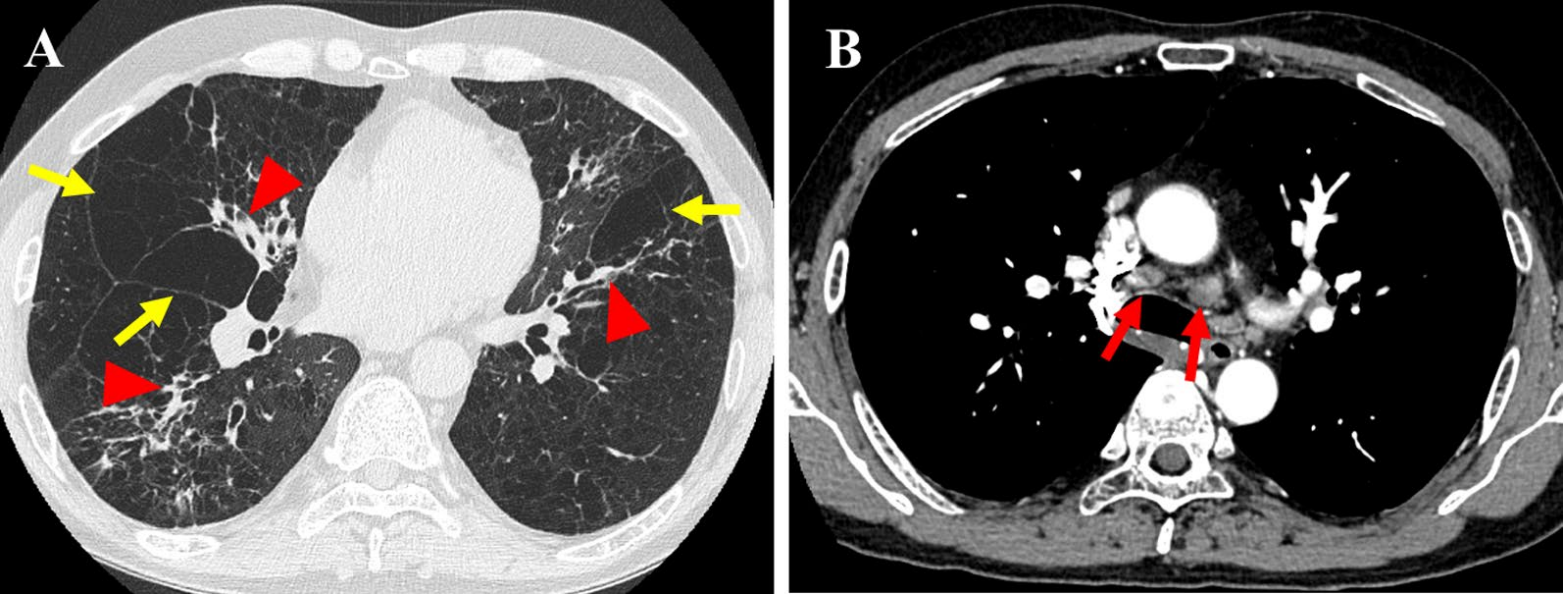
Preoperative chest computed tomography revealed thickening of the bronchovascular bundles (red arow head), ground-glass opacities, scattered cystic lesions (yellow arrow) (**A**), and slight enlargement of the mediastinal lymph nodes (red arrow) (**B**)

**Fig. 2:**
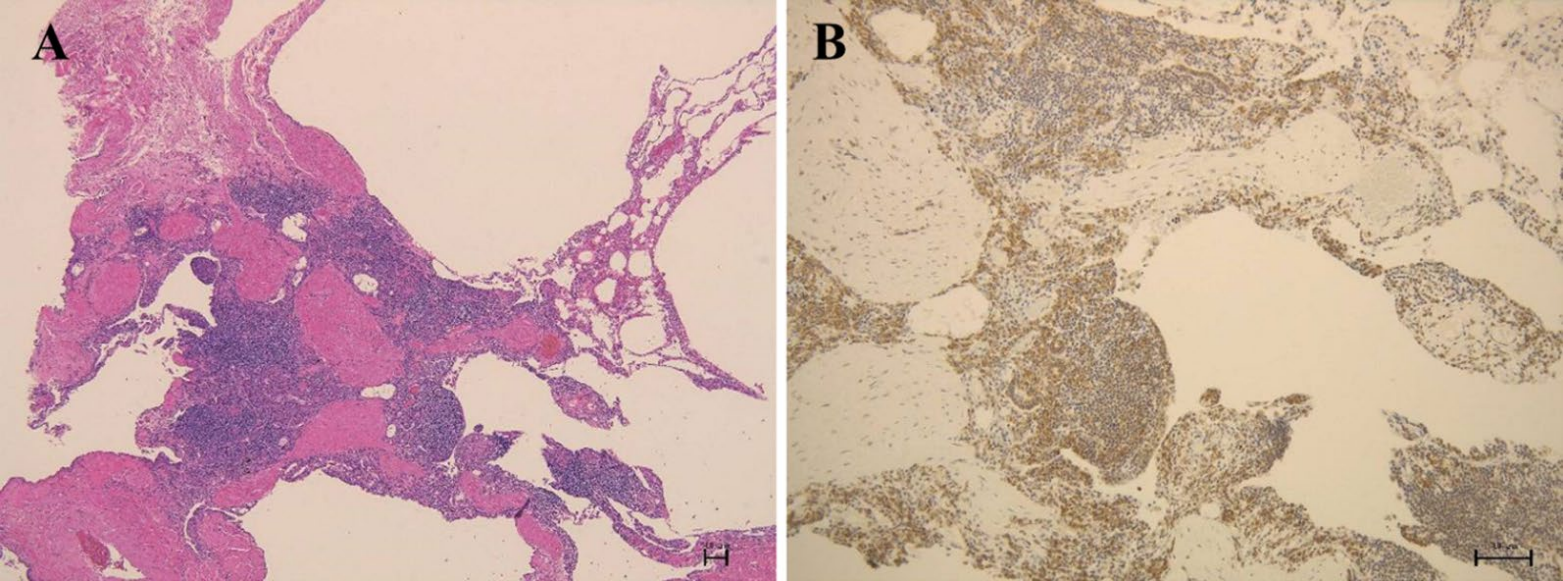
Pathology of the explanted native right lung. **A** Lower lobe shows a cystic enlargement of the alveolar walls and interstitial infiltration of lymphocytes and plasma cells (hematoxylin and eosin staining, original magnification: 40 ×). **B** Lower lobe shows infiltration of plasma cells with interleukin-6 (IL-6)-positivity (Immunofluorescence staining of IL-6, original magnification: 100 ×)

**Fig. 3:**
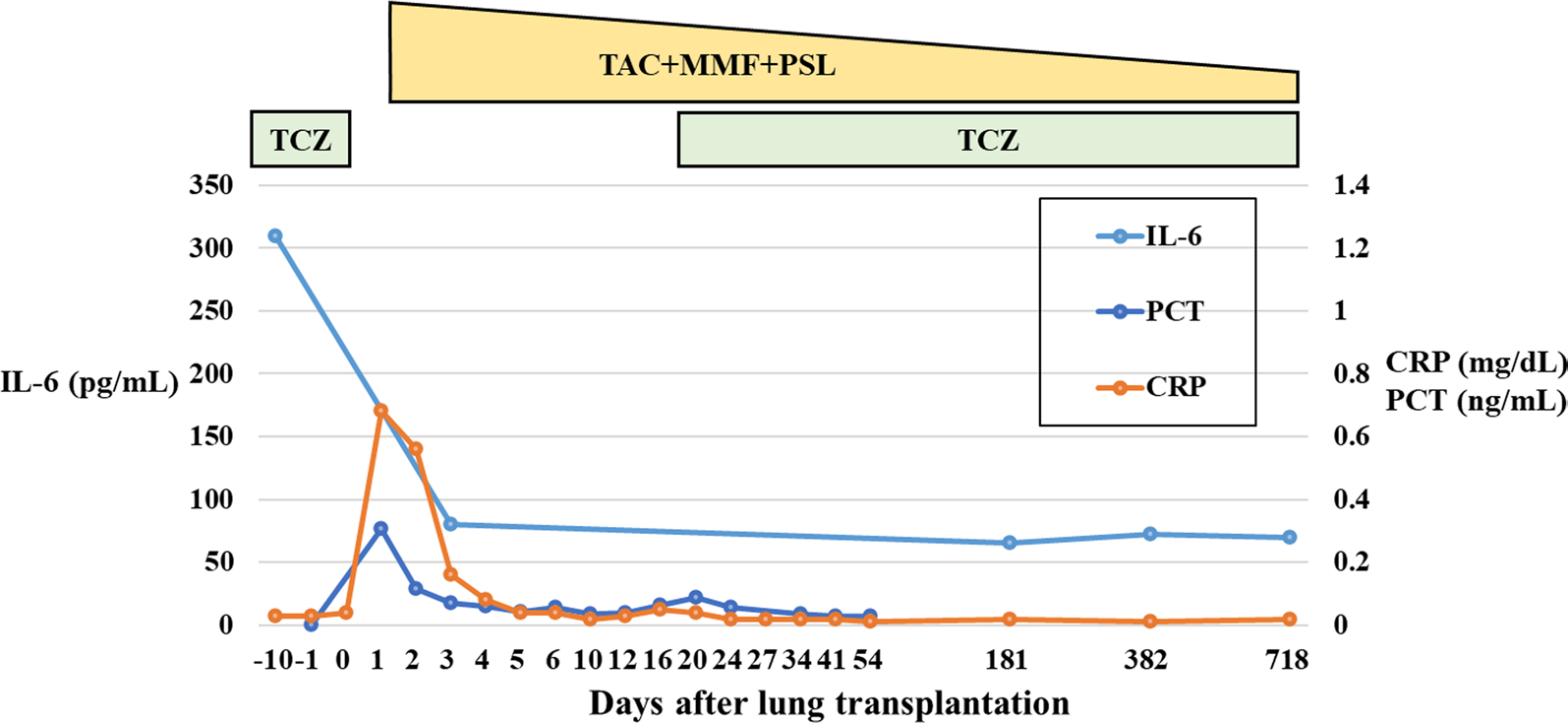
Postoperative course after lung transplantation. Tapering of triple immunosuppression (tacrolimus [TAC], mycophenolate mofetil [MMF], and prednisolone [PSL]) and sustained treatment with tocilizumab (TCZ) in an idiopathic multicentric Castleman disease transplant recipient. The interleukin-6 (IL-6) levels are maintained at 60–80 pg/mL, and the C-reactive protein (CRP) and procalcitonin (PCT) levels are trending towards normal with no allograft rejection

A 30-year-old man with fever, anemia and slight enlargement of the mediastinal lymph nodes was diagnosed with iMCD via lymph node biopsy. Laboratory tests revealed elevated levels of serum total protein (11.1 g/dL), serum IL-6 (44.5 pg/mL), and immunoglobulin G (IgG) (3870 mg/dL). Since treatment with oral prednisolone (PSL) failed to suppress the symptoms of iMCD, intravenous TCZ (8 mg/kg, every 2 weeks) was administered. Although oral PSL with intravenous TCZ had controlled his iMCD symptoms without the further exacerbation of his lung disease, his respiratory function continued to decline gradually, and he was registered for cadaveric LTx at 46 years of age (Fig. 1). He required oxygen therapy at home throughout the day. IL-6 levels remained at 200–350 pg/ml after treatment with intravenous TCZ. At 48 years of age, he underwent right single-LTx from a cadaveric donor. The entire transplant process was uneventful, with slight pleural adhesions and slight enlargement of the hilar lymph nodes. Histological examination of the resected lung revealed iMCD (Fig. 2). Postoperative immunosuppression included a usual triple-drug regimen (tacrolimus, mycophenolate mofetil, and PSL). TCZ was discontinued once after LTx to prevent overimmunosuppression. The postoperative course was uneventful, and there was no infection or rejection. Serum IL-6 levels were maintained at a relatively low level (80 pg/mL) (Fig. 3), and postoperative serum IgG levels had decreased. C-reactive protein (CRP) and procalcitonin (PCT) levels were not significantly elevated in the perioperative period (Fig. 3). At the time of the preparation of this report, 32 months after LTx, the patient remained asymptomatic, including pulmonary manifestations, and the allograft function was preserved without evidence of rejection or recurrent iMCD. In addition, laboratory tests did not demonstrate any significant adverse effects of TCZ (such as dyslipidemia or myelosuppression), cytomegalovirus infection events, or development of de novo donor-specific antibody (DSA). As IL-6 levels remained at 60–80 pg/mL (Fig. 3), the patient received intravenous TCZ treatment (8 mg/kg, every 2 weeks) under immunosuppression with a triple-drug regimen to preserve the allograft lung.

## Discussion

We have identified two important clinical implications. Cadaveric single-LTx for ESLD secondary to iMCD can be a possible therapeutic option, and TCZ can alleviate iMCD, ensuring a stable perioperative period during and after LTx.

First, LTx is an acceptable therapeutic option for patients with iMCD and ESLD. It is generally challenging to determine an indication for LTx for the treatment of ESLD secondary to iMCD due to its characterization as a systemic disease with possible multiple organ involvement. Although lung involvement in iMCD is common, to the best of our knowledge, there have been only two reports of LTx for the treatment of ESLD secondary to iMCD [4, 5]. One of the reports with 6 month follow-up showed that single-LTx can be feasible if the clinical manifestations of iMCD, except for the lungs, are controlled [5]. In our case, in an iMCD patient with bilateral lung involvement, single-LTx was an effective treatment without any complications in the residual native lung with a follow-up period of 32 months. However, since iMCD may recur with cessation of TCZ, lifelong administration of TCZ may be necessary to protect the allograft lung and control iMCD disease activity after transplantation, regardless of the procedure of single- or bilateral-LTx [8].

Second, TCZ, in addition to standard immunosuppressive therapy using a triple-drug regimen (tacrolimus, mycophenolate mofetil, and PSL), has been useful for iMCD during and after LTx. In this case, the IL-6 level was maintained at a low level (< 100 pg/mL), even with a temporal cessation of TCZ for 28 days after LTx. Glucocorticoid use following LTx suppresses hypercytokinemia and alleviates symptoms [2], and tacrolimus prevents iMCD from worsening by suppressing inflammation of T-helper 1 cells, which is considered a pathogenesis of iMCD [9]. Furthermore, the patient experienced no apparent rejection or development of DSA during the observation period. IL-6, a major cytokine involved in the transition of B cells to IgG-secreting plasma cells and finally to plasmacytoid cells, also stimulates Th17 cells which cause inflammation and graft rejection. Recent studies suggest that TCZ inhibits antibody production and suppresses inflammation by targeting the IL-6/IL-6R pathway [10, 11]. In the field of renal transplantation, it has been reported that patients with chronic antibody-mediated rejection who failed other therapies were treated with TCZ and had a significant decrease in DSA and stable renal function after 2 years [12, 13]. Thus, TCZ can reduce the risk of chronic antibody-mediated rejection after LTx.

Notably, in this case, the serum CRP level was not significantly elevated in the perioperative period. Although CRP levels are usually elevated even in patients without signs of sepsis and who are on immunosuppressive drugs [14], suppression of CRP in patients treated with TCZ can delay the diagnosis of serious infections [15]. It should be noted that CRP levels can be elevated in the perioperative period in lung transplant patients receiving TCZ. PCT, which is a marker of bacterial infection produced by a pathway independent of IL-6, has been proposed as a preferred surrogate marker of bacterial infection during TCZ treatment [16].

## Conclusion

Single-LTx can be a feasible treatment option for ESLD due to iMCD, and TCZ in combination with usual immunosuppression can be helpful in stabilizing iMCD patients pre-and post-LTx.

## Data Availability

Not applicable.

## Abbreviations

CRP: C-reactive protein
CT: Computed tomography
DSA: Donor-specific antibody
ESLD: End-stage lung disease
IgG: Immunoglobulin G
IL-6: Interleukin-6
iMCD: Idiopathic multicentric Castleman disease
LTx: Lung transplantation
PCT: Procalcitonin
PSL: Prednisolone
TCZ: Tocilizumab

## References

[1] Fajgenbaum DC, Uldrick TS, Bagg A, Frank D, Wu D, Srkalovic G (2017). International, evidence-based consensus diagnostic criteria for HHV-8-negative/idiopathic multicentric Castleman disease. Blood.

[2] Liu AY, Nabel CS, Finkelman BS, Ruth JR, Kurzrock R, van Rhee F (2016). Idiopathic multicentric Castleman’s disease: a systematic literature review. Lancet Haematol.

[3] Nishimoto N, Kanakura Y, Aozasa K, Johkoh T, Nakamura M, Nakano S (2005). Humanized anti-interleukin-6 receptor antibody treatment of multicentric Castleman disease. Blood.

[4] Chin AC, Stich D, White FV, Radhakrishnan J, Holterman MJ (2001). Paraneoplastic pemphigus and bronchiolitis obliterans associated with a mediastinal mass: a rare case of Castleman’s disease with respiratory failure requiring lung transplantation. J Pediatr Surg.

[5] Morimura Y, Chen F, Kinjo T, Miyagawa-Hayashino A, Kubo T, Yamada T (2014). Successful single-lung transplantation for multicentric Castleman disease. Ann Thorac Surg.

[6] Nishimoto N, Sasai M, Shima Y, Nakagawa M, Matsumoto T, Shirai T (2000). Improvement in Castleman’s disease by humanized anti-interleukin-6 receptor antibody therapy. Blood.

[7] Nishimoto N, Terao K, Mima T, Nakahara H, Takagi N, Kakehi T (2008). Mechanisms and pathologic significances in increase in serum interleukin-6 (IL-6) and soluble IL-6 receptor after administration of an anti-IL-6 receptor antibody, tocilizumab, in patients with rheumatoid arthritis and Castleman disease. Blood.

[8] Akiyama M, Yasuoka H, Takeuchi T (2017). Interleukin-6 in idiopathic multicentric Castleman's disease after long-term tocilizumab. Ann Hematol.

[9] Shirai T, Onishi A, Waki D, Saegusa J, Morinobu A (2018). Successful treatment with tacrolimus in TAFRO syndrome: two case reports and literature review. Medicine (Baltimore).

[10] Jordan SC, Choi J, Vo A (2015). Kidney transplantation in highly sensitized patients. Br Med Bull.

[11] Vo AA, Choi J, Kim I, Louie S, Cisneros K, Kahwaji J (2015). A Phase I/II trial of the interleukin-6 receptor-specific humanized monoclonal (tocilizumab) + intravenous immunoglobulin in difficult to desensitize patients. Transplantation.

[12] Choi J, Aubert O, Vo A, Loupy A, Haas M, Puliyanda D (2017). Assessment of tocilizumab (anti-interleukin-6 receptor monoclonal) as a potential treatment for chronic antibody-mediated rejection and transplant glomerulopathy in HLA-sensitized renal allograft recipients. Am J Transplant.

[13] Jordan SC, Choi J, Kim I, Wu G, Toyoda M, Shin B (2017). Interleukin-6, A cytokine critical to mediation of inflammation, autoimmunity and allograft rejection: therapeutic implications of IL-6 receptor blockade. Transplantation.

[14] Kroesen S, Widmer AF, Tyndall A, Hasler P (2003). Serious bacterial infections in patients with rheumatoid arthritis under anti-TNF-alpha therapy. Rheumatology (Oxford).

[15] Nguyen MT, Pødenphant J, Ravn P (2013). Three cases of severely disseminated Staphylococcus aureus infection in patients treated with tocilizumab. BMJ Case Rep.

[16] Gaensbauer JT, Press CA, Hollister JR, Asturias EJ (2013). Procalcitonin: a marker of infection not subverted by treatment with interleukin-6 receptor inhibition. Pediatr Infect Dis J.

